# The complete chloroplast genome of *Solanum sisymbriifolium* (Solanaceae), the wild eggplant

**DOI:** 10.1080/23802359.2022.2077667

**Published:** 2022-05-26

**Authors:** Mengying Yin, Yanan Yu, Yaju Gong, Min Gui, Zhibin Li, Rui Bao, Jie Cheng, Guanghui Du, Liyan Wu

**Affiliations:** a Institute of Resource Plants, Yunnan University, Kunming, China; b Horticultural Research Institute, Yunnan Academy of Agricultural Sciences, Kunming, China

**Keywords:** *Solanum sisymbriifolium*, chloroplast genome, phylogenetic tree

## Abstract

*Solanum sisymbriifolium* is a critical wild eggplant resource with resistance to many serious diseases that affect eggplant production. In this study, the chloroplast genome of *S. sisymbriifolium* was successfully sequenced using Illumina high-throughput sequencing technology. The length of the complete chloroplast genome is 155,771 bp, and its GC content is 37.76%. There is a large single-copy region (86,404 bp), a small single-copy region (18,525 bp), and a pair of inverted repeat regions (25,421 bp) in the chloroplast genome. A total of 128 coding genes were annotated in the entire chloroplast genome, including 83 protein-coding genes, 37 transfer RNA genes and eight ribosomal RNA genes. The phylogenetic tree of 17 complete chloroplast genomes shows that *S. sisymbriifolium* is closely related to *Solanum wrightii*.

*Solanum sisymbriifolium* (*Solanum sisymbriifolium* Lamarck 1758), a native of South America, is a perennial herbaceous plant belonging to the genus *Solanum* of Solanaceae. Previously, it had been cultivated in Guangdong and Yunnan provinces in China, and now it grows in the wild in Kunming (Chinese Botanical Society Editorial Board of Chinese Academy of Sciences 1978). In recent years, studies have shown that *S. sisymbriifolium* has resistance against many serious diseases and pests of Solanaceae (Collonnier et al. 2001), especially verticillium wilt (Fassuliotis and Dukes 1972; Wu et al. 2019), phomopsis blight (Kalda et al. 1977 ), bacterial wilt (Mochizuki and Yamakawa 1979) and nematodes (Fassuliotis and Dukes 1972; Dias et al. 2012). To realize its potential for providing disease resistance, *S. sisymbriifolium* could be used as an important rootstock for tomato production (Baidya et al. 2017; Deb et al. 2019); it has also been used as a trap crop for potato cyst nematodes (Timmermans et al. 2009; Dias et al. 2017). However, although transcriptome-related research has been carried out (Wu et al. 2019), there is no genomic information on *S. sisymbriifolium*, which significantly limits its utilization and related research. Here, the complete chloroplast genome of *S. sisymbriifolium* is reported, providing genomic data for the phylogenetic analysis of the genus *Solanum*. Importantly, the results will lay a foundation for conservation genetics and molecular research on this plant.

In this study, fresh leaves of *S. sisymbriifolium* were collected from the Horticultural Institute of Yunnan Academy of Agricultural Sciences (25°7′27″N, 102°45′46″E), Kunming, China. The specimens was deposited at the Herbarium of Kunming Institute of Botany of CAS (http://www.kun.ac.cn, Xuedan Xie and xiexuedan@mail.kib.ac.cn) under the specimen code: KUN184762. A modified CTAB method (Yang et al. 2014) was used to extract high-quality total genomic DNA. The quality and quantity of the extracted DNA were examined using a NanoDrop 2000 spectrophotometer (NanoDrop Technologies, Wilmington, DE, USA), Qubit dsDNA HS Assay Kit on a Qubit 3.0 Fluorometer (Life Technologies, Carlsbad, CA, USA) and electrophoresis on a 0.8% agarose gel. Then, the genomic DNA was sent to Shanghai Majorbio Biopharm Technology Company (Shanghai, China) for sequencing by Illumina NovaSeq. Raw reads were filtered by using the NGSQC toolkit with default parameters to obtain clean reads of high quality (Patel and Jain 2012). The clean reads were trimmed and assembled by NOVOPlasty software (Dierckxsens et al. 2017). Then, the assembled sequences were analyzed for possible assembly errors by collinearity with related species using Mummer (http://mummer.sourceforge.net/manual/). Finally, the assembled chloroplast genome was annotated by PGA (Qu et al. 2019).

The size of the complete chloroplast genome of *S. sisymbriifolium* (GenBank accession number: OL597592) is 155,771 bp, and its overall GC content is 37.76%. The chloroplast genome had a characteristic quadripartite circular structure, and it was comprised of a large single-copy region (86,404 bp), a small single-copy region (18,525 bp), and a pair of inverted repeat regions (25,421 bp). In addition, there were 83 protein-coding genes, 37 transfer RNA (tRNA) genes, and 8 ribosomal RNA (rRNA) genes in the entire genome.

To explore the phylogenetic relationship of *S. sisymbriifolium* in *Solanum*, 15 complete chloroplast genomes of *Solanum* species were used to construct the phylogenetic tree, and 2 species (*Capsicum annuum* and *Solanum lycopersicum*) in Solanaceae were selected as an outgroup. These 17 published sequences were obtained from NCBI GenBank. All chloroplast genome sequences were aligned using MAFFT software (Katoh and Standley 2013). With 100 bootstrap replicates, a neighbor-joining phylogenetic tree was constructed by MEGA X software. The phylogenetic analysis results showed that *S*. *sisymbriifolium* was most closely related to *Solanum wrightii* among the other 16 species (Figure 1). Together, these results will provide a reference for future studies of *Solanum* chloroplasts.

**Figure 1.**
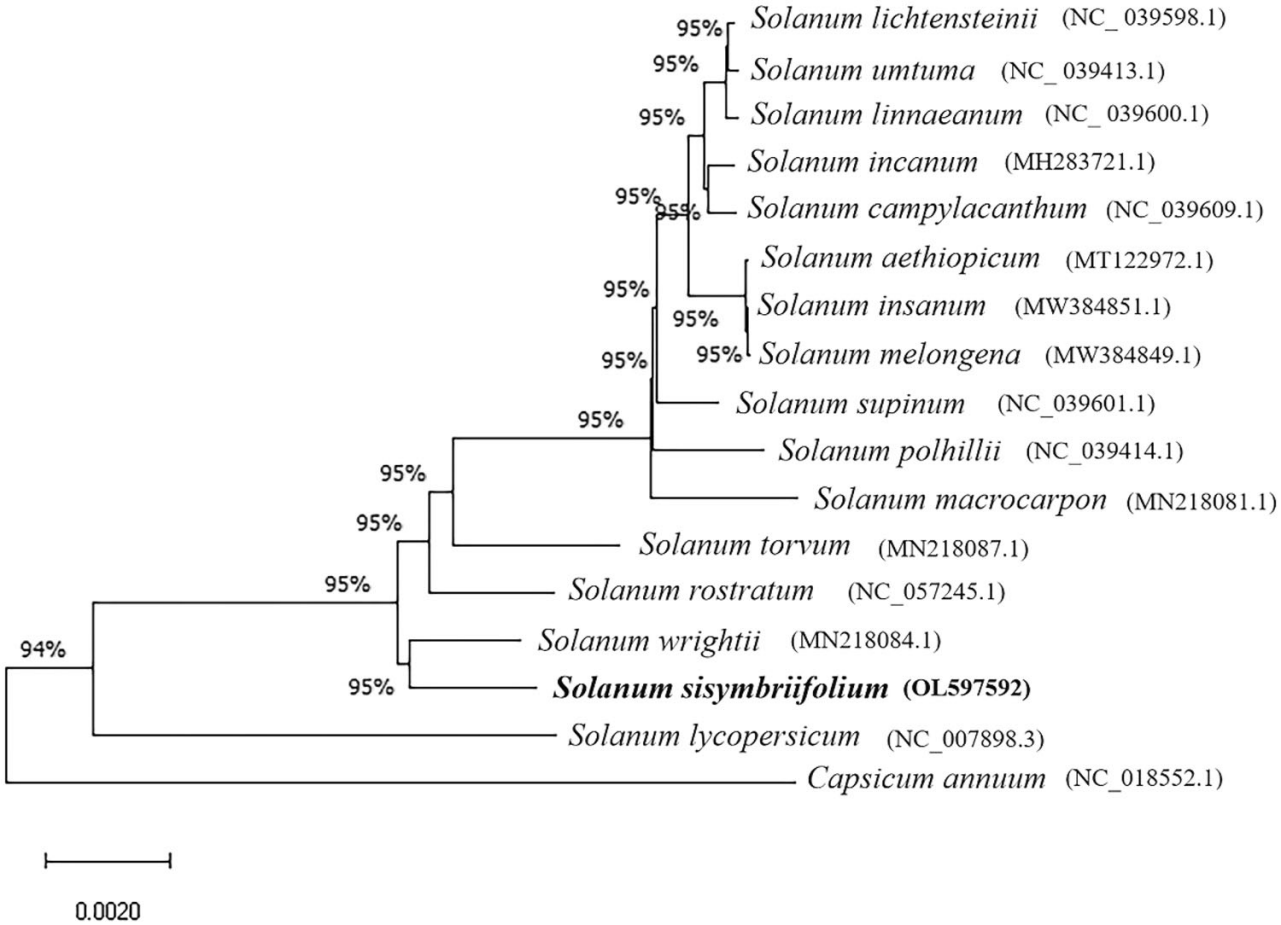
Phylogenetic tree of 17 complete chloroplast genomes.

## Ethical approval

The research on plants in this study, including the collection of plant materials, has been carried out in accordance with guidelines provided by the author's institution and national or international regulations.

## Author contributions

M.Y.Y. and Y.N.Y. conceived and designed the research framework, and drafted the manuscript. Y.J.G., M.G., and Z.B.L. analyzed the data. R.B. and J.C. cultivated the seedlings and helped with sampling. G.H.D. and L.Y.W. conceived the study, participated in its design and coordination, and helped draft the manuscript. All authors read and approved the final manuscript.

## Data Availability

The genome sequence data that support the findings of this study are openly available in GenBank of NCBI at https://www.ncbi.nlm.nih.gov, reference number OL597592. The associated BioProject, SRA, and Bio-Sample numbers are PRJNA809910, SRR18136644, and SAMN26209660, respectively.

## References

[CIT0001] Baidya S, Timila RD, Kc RB, Manandhar HK, Manandhar C. 2017. Management of root knot nematode on tomato through grafting root stock of *Solanum sisymbriifolium*. J Nep Agric Res Counc. 3:886–31.

[CIT0002] Chinese Botanical Society Editorial Board of Chinese Academy of Sciences. 1978. Flora of China. Beijing: Science Press; p. 113.

[CIT0003] Collonnier C, Fock I, Kashyap V, Rotino GL, Daunay MC, Lian Y, Mariska IK, Rajam MV, Servaes A, Ducreux G, et al. 2001. Applications of biotechnology in eggplant. Plant Cell Tiss Org. 65(2):91–107.

[CIT0004] Deb G, Bhuiyan MSU, Sarker KK, Papry AS, Sultana S. 2019. *In vitro* plant regeneration of wild eggplant (*Solanum sisymbriifolium*) to produce large number of rootstocks for tomato grafting. J Adv Biotechnol Exp Ther. 2(2):65–72.

[CIT0005] Dias MC, Conceição IL, Abrantes I, Abrantes I, Cunha MJ. 2012. *Solanum sisymbriifolium* – a new approach for the management of plant-parasitic nematodes. Eur J Plant Pathol. 133(1):171–179.

[CIT0006] Dias MC, Perpétuo LS, Cabral AT, Cabral AT, Guilherme R, da Cunha MJM, Melo F, Machado ÓC, Conceição IL. 2017. Effects of *Solanum sisymbriifolium* on potato cyst nematode populations in Portugal. Plant Soil. 421(1–2):439–414.

[CIT0007] Dierckxsens N, Mardulyn P, Smits G. 2017. NOVOPlasty: *de novo* assembly of organelle genomes from whole genome data. Nucleic Acids Res. 45(4):e18. 28204566 10.1093/nar/gkw955 PMC5389512

[CIT0008] Fassuliotis G, Dukes PD. 1972. Disease reaction of *Solanum melongena* and *S. sisymbrifolium* to *Meloidogyne incognita* and *Verticillium alboatrum*. J Nematol. 4(4):222–223.

[CIT0016] Kalda TS, Swarup V, Choudhury B. 1977. Resistance to Phomopsis blight in eggplant. Veg Sci. 4:90–101.

[CIT0009] Katoh K, Standley DM. 2013. MAFFT multiple sequence alignment software version 7: improvements in performance and usability. Mol Biol Evol. 30(4):772–780. 23329690 10.1093/molbev/mst010 PMC3603318

[CIT0010] Mochizuki H, Yamakawa K. 1979. Resistance of selected eggplant cultivars and related wild species to bacterial wilt (*Pseudomonas solanacearum*). Bull Veg Ornem Crop Res Stn. 6:1–10.

[CIT0011] Patel RK, Jain M. 2012. NGS QC toolkit: a toolkit for quality control of next generation sequencing data. PLOS One. 7(2):e30619. 22312429 10.1371/journal.pone.0030619 PMC3270013

[CIT0012] Qu XJ, Moore MJ, Li DZ, Yi TS. 2019. PGA: a software package for rapid, accurate, and flexible batch annotation of plastomes. Plant Methods. 15:50. 31139240 10.1186/s13007-019-0435-7 PMC6528300

[CIT0013] Timmermans B, Vos J, Stomph TJ. 2009. The development, validation and application of a crop growth model to assess the potential of *Solanum sisymbriifolium* as a trap crop for potato cyst nematodes in Europe. Field Crop Res. 111(1–2):22–31.

[CIT0014] Wu LY, Du GH, Bao R, Li ZB, Gong YJ, Liu FH. 2019. *De novo* assembly and discovery of genes involved in the response of *Solanum sisymbriifolium* to *Verticillium dahlia*. Physiol Mol Biol Plants. 25(4):1009–1027. 31402823 10.1007/s12298-019-00666-4 PMC6656901

[CIT0015] Yang JB, Li DZ, Li HT. 2014. Highly effective sequencing whole chloroplast genomes of angiosperms by nine novel universal primer pairs. Mol Ecol Resour. 14(5):1024–1031. 24620934 10.1111/1755-0998.12251

